# Pancreas transplantation: a single-institution experience in Japan

**DOI:** 10.1007/s00595-013-0516-6

**Published:** 2013-02-20

**Authors:** Yasuhiro Okabe, Hidehisa Kitada, Yoshifumi Miura, Takehiro Nishiki, Kei Kurihara, Sayako Kawanami, Soshi Terasaka, Keizo Kaku, Hiroshi Noguchi, Atsushi Sugitani, Masao Tanaka

**Affiliations:** 1 Department of Surgery and Oncology, Graduate School of Medical Sciences, Kyushu University, 3-1-1 Maidashi, Higashi-ku, Fukuoka, 812-8582 Japan; 2 Present Address: Department of Organ Transplantation and Regenerative Medicine, Fujita Health University, Toyoake, Japan

**Keywords:** Pancreas transplantation, Venous thrombosis, Risk factor

## Abstract

**Purpose:**

We herein report our experience with pancreas transplantation in 26 patients at a single institution in Japan between August 2001 and December 2011.

**Methods:**

We reviewed the medical records of 26 pancreas transplantations performed in our institute.

**Results:**

The early complications (within 2 weeks) included one graft venous thrombosis, one arterial thrombosis, and two reoperations for bleeding. Of the 26 pancreas transplant recipients, five lost pancreas graft function. Of 24 simultaneous pancreas–kidney recipients, three lost kidney graft function due to noncompliance. The patient, pancreas, and kidney survival rates were 100, 96 and 93 % at 1 year; 100, 80 and 93 % at 5 years; and 100, 67 and 68 % at 10 years, respectively. Of all these complications, venous thrombosis after pancreas transplantation was the most critical.

**Conclusions:**

As the largest series of pancreas transplantations in a single institution in Japan, our series yielded better results than the worldwide data recorded by the International Pancreas Transplant Registry. Routine postoperative anticoagulation therapy is not necessary for the prevention of graft thrombosis if sufficient fluid infusion is strictly controlled and the graft blood flow is frequently monitored. When graft thrombosis occurs, both early detection and appropriate intervention are extremely important if the pancreas graft is to survive.

## Introduction

In patients with type 1 diabetes, pancreas transplantation normalizes the glucose metabolism, prevents the progression of secondary complications, and greatly improves the quality of life. About 35,000 pancreas transplantations had already been performed worldwide by 2010 [1]. Since enforcement of the Organ Transplant Law in October 1997, the number of organ transplantations has been increasing in Japan, and 121 pancreas transplantations had been carried out by the end of 2011 (unpublished data reported by the Japanese Pancreas and Islet Transplantation Association). We had performed 26 pancreas transplantations at our institution as of December 2011. Although this number is small, this is the largest series of pancreas transplantations to be reported in a single institution in Japan.

Pancreas graft survival has been improving with the development of immunosuppressive drugs and improvements in postoperative management. However, certain complications still may occur, especially during the early postoperative period. Postoperative complications such as thrombosis, bleeding, infection, anastomotic leakage, and pancreatitis can lead to graft loss. In this study, we retrospectively review our results of pancreas transplantation, with a special emphasis on postoperative complications, including a case of venous thrombus that occurred on the ninth day after simultaneous pancreas–kidney transplantation (SPK), which resulted in graft loss (3.8 %). Although the number of patients reported is small in terms of pancreas transplantation, this is the largest experience of pancreas transplantation in Japan at the present time.

## Methods

Twenty-six recipients (7 males and 19 females) who underwent pancreas transplantation at Kyushu University Hospital from August 2001 to December 2011 were enrolled in this study. All patients had type 1 diabetes. Of these recipients, 19 underwent SPK, two underwent pancreas transplantation alone (PTA), one underwent pancreas after kidney transplantation (PAK) from brain-dead donors (BDD), and two underwent SPK from non-heart-beating donors (NHBD). We also performed living-donor SPK in two patients with severe and life-threatening hypoglycemic attacks (Table 1).

**Table 1:** Types of pancreas transplantation performed at our institution

Type of transplant and donor	Number
SPK from BDD	19
SPK from NHBD	2
PAK from BDD	1
PTA from BDD	2
LSPK	2

*SPK* simultaneous pancreas–kidney transplantation, *PAK* pancreas after kidney transplantation, *PTA* pancreas transplantation alone, *BDD* brain-dead donor, *NHBD* non-heart-beating donor, *LSPK* living-donor SPK

The immunosuppressive protocols included calcineurin inhibitors (tacrolimus/cyclosporine), mycophenolate mofetil, steroids, and basiliximab induction. Anti-thymoglobulin could not be employed for induction therapy in Japan until March 2012. We did not routinely use heparin after the operation. Instead, ultrasonography was frequently performed for early detection of thrombosis of the pancreas/kidney grafts or a bleeding episode. The ultrasonographic apparatus consisted of a model LOGIQ 7 system (GE Healthcare, Tokyo, Japan) and a 4- to 5.5-MHz convex probe for color Doppler and B-FLOW imaging. The induction of dialysis, continuous use of insulin, and re-transplantation were counted as graft loss. The survival rates of patients, pancreas grafts and kidney grafts were calculated using the Kaplan–Meier method. We used the JMP software program, version 9 for Windows.

The evaluated donor risk factors predisposing the recipients to thrombosis included age, cause of death, use of a vasopressor agent, body mass index (BMI), use of desmopressin, pancreas preservation time, cardiac arrest, and resuscitation time. The evaluated operation-related risk factors included graft artery reconstruction, vein extension, enteric drainage of pancreatic juice, and the use of left iliac vessels for pancreas transplantation. The evaluated recipient risk factors included the need for preoperative dialysis, BMI, age, and duration of diabetes (Table 2) [4–12]. We compared the case of graft venous thrombosis and the other 25 cases according to the risk factors for venous thrombosis reported from various institutions [4–12] (Table 3), and we carefully considered the postoperative management of our department, especially for venous thrombosis after pancreas transplantation.

**Table 2:** Risk factors for thrombosis after pancreas transplantation

Donor factors
Age >45 years Cause of death: cardiac and cerebrovascular disease Poor hemodynamic state, use of vasopressors BMI >30 kg/m^2^ Use of desmopressin
Operation-related factors
Complicated arterial reconstructions Extension of vein Left side transplant Partial pancreas transplantation
Recipient factors
No induction of preoperative HD Obesity No use of heparin Postoperative graft pancreatitis High dose of IVIG BMI >30 kg/m^2^ Age Duration of diabetes

*BMI* body mass index, *HD* hemodialysis, *IVIG* intravenous immunoglobulin therapy

**Table 3:** Comparison between the graft venous thrombus case and the other 25 cases

Venous thrombus case	The other 25 cases
Donor factors	
Age: 26 years	Average age: 38.8 years
Cause of death: non-cardiac/CVD	Cardiac and CVD: 13/25
Vasopressor: dopamine + NAD: 2 agents	One agent: 14/25; 2+ agents: 9/25
Donor BMI: 19.4 kg/m^2^	Average BMI: 23.0 kg/m^2^
Desmopressin: (+)	Desmopressin use: 19/25
Pancreas preservation time: 831 min	Average time: 784.6 min
Non-heart beating donor: (−)	Non-heart beating donor: 2/25
Cardiac arrest: (+)	Cardiac arrest: 9/25
Resuscitation time: 47 min	Resuscitation time: 39.8 min
Operation-related factors	
Artery reconstruction: Carrel patch + CHA-I graft (−)	Y graft: 4/25 CHA-I graft: 6/25
Vein extension: (−)	Vein extension: 6/25
Pancreatic juice drainage: enteric	Enteric drainage: 20/25
Left side transplant: (−)	Left side transplant: 1/25 (PAK case)
Partial pancreas transplant: (−)	Partial pancreas transplant from living donor: 2/25
Recipient factors	
Preoperative hemodialysis: (+)	No hemodialysis before transplant: 3/25
Recipient BMI: 27.2 kg/m^2^	Average BMI: 20.1 kg/m^2^
Age: 33 years	Age: 38.9 years
Duration of diabetes: 19 years	Duration of diabetes: 23.4 years

*CVD* cerebrovascular disease, *NAD* noradrenalin, *BMI* body mass index, *CHA* common hepatic artery

## Results

The patient, pancreas graft, and kidney graft survival rates were 100, 96 and 100 % at 1 year; 100, 80 and 93 % at 5 years; and 100, 67 and 68 % at 10 years, respectively. Complications requiring reoperations included venous thrombosis and graft loss (*n* = 1), postoperative bleeding (*n* = 2), arterial thrombosis (*n* = 1), intestinal obstruction (*n* = 1), and bladder-to-enteric drainage conversion due to bleeding from the graft duodenum requiring duodenectomy (*n* = 1). Among the 26 pancreas transplant recipients, five pancreas grafts were lost due to venous thrombosis on postoperative day (POD) 9 in one patient, recurrent insulin-dependent diabetes mellitus caused by autoimmune isletitis on POD 2560 in one patient, noncompliance on PODs 519 and 1195 in two patients, and rejection on POD 730 in one patient. Of the 23 pancreas–kidney transplant recipients, three kidney grafts were lost, all of which were due to noncompliance (on PODs 1693, 1855 and 2704).

As indicated above, we experienced one patient with venous thrombosis that caused pancreas graft loss. At 26 years of age, the BDD of the patient with venous thrombosis was younger than that of the others. The cause of death of the BDD was not a risk factor. During the perioperative management of the BDD, two vasopressor agents were used; desmopressin was also administered. However, in the other 23 deceased donors, more than two vasopressor agents were used in nine patients, and desmopressin was given to 19 patients. The pancreas preservation time was 831 min, which was not significantly different from the others durations. Although cardiopulmonary resuscitation was performed for 47 min after cardiac arrest, nine other donors also had developed cardiac arrest, and the average resuscitation time was 39.8 min. Therefore, no major differences were observed in the donor factors between the recipient who developed venous thrombosis and the others. Moreover, two patients with SPK transplants from NHBD did not develop venous thrombosis.

Regarding operation-related factors, graft venous thrombosis occurred in one recipient without complicated arterial reconstruction or portal vein extension, although we employed various methods for vascular reconstruction, such as Y grafts, I grafts of the common hepatic-gastroduodenal artery, and portal vein extension. Enteric pancreatic drainage, thought to be a risk factor, was chosen in this recipient, but the same drainage was used in 20 of 23 recipients in the SPK group. Left-sided pancreas transplantation and the use of a partial pancreas are also considered to be risk factors. However, these were not used in the recipient with venous thrombosis. There were thus no significant operation-related factors that were believed to have increased the risk of venous thrombosis in this recipient.

In terms of the recipient factors, the presence of preoperative dialysis and the higher BMI of the recipient (27.2 kg/m^2^) compared with the mean BMI of the others (20.1 kg/m^2^) were significant among the various risk factors for thrombosis (Table 2).

**Fig. 1:**
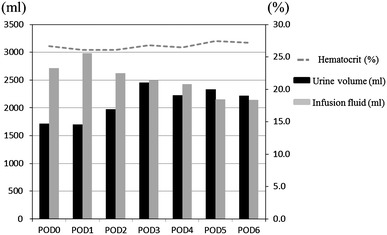
Hematocrit level, urine volume and fluid infusion in 24 recipients of a whole pancreas graft

The average hematocrit, urine volume, and fluid infusion volume in the 24 patients who underwent whole pancreas transplantation are shown in Fig. 1. The average hematocrit on POD 6 was 27.3 %.

## Discussion

Pancreas transplantation improves not only quality of life, but also the survival, of patients with type 1 diabetes. Pancreas graft survival has been improving following the recent developments in immunosuppressive drugs and improvements in surgical techniques and postoperative management. In patients with type 1 diabetes on hemodialysis and on the waiting list, the 5-year survival is <50 %. Japanese recipient candidates on the waiting list tend to be older and have a longer diabetic and/or hemodialysis period. In addition, many donors are considered to be marginal according to Kapur’s criteria [15]. In this respect, our data show relatively good results compared with other reports [1–3]. However, organ shortage is a serious problem in Japan. Pancreas transplantation has been performed in only 121 patients in the last 12 years nationwide. Therefore, the waiting period is getting longer, with a smaller chance of transplantation than in other countries. For these reasons, special attention must be paid to prevent early graft loss. The major causes of pancreas graft loss are usually technical failures in the early period after transplantation. In particular, graft venous thrombosis frequently leads to graft loss.

Certain postoperative complications, such as thrombosis, bleeding, infection, anastomotic leakage and pancreatitis, may occur after pancreas transplantation. Moreover, patients with type 1 diabetes and end-stage renal failure are reportedly in a hypercoagulable state, which is one of the causes of susceptibility to thrombosis after pancreas transplantation [13]. It was previously shown that ischemia/reperfusion injury makes the graft locally hypercoagulable [14]. Furthermore, compared with the USA and Europe, many Japanese donors are marginally adequate for transplantation. Of 61 donors previously reported in this country, 45 (73.8 %) were marginal donors according to Kapur’s definition, including (1) donors aged ≥45 years, (2) donors with an unstable hemodynamic state, and (3) NHBDs [15]. These factors may have led to the increased rate of perioperative complications in the recipients. The reported rate of graft venous thrombosis after pancreas transplantation at various institutions is 1–13 % [4, 10, 16–19]. The United Network for Organ Sharing (UNOS) reported in 2004 that the rate of graft venous thrombosis was 7.8 % in PTA, 5.3 % in PAK, and 4.9 % in SPK [11]. Graft venous thrombosis occurred in only one case of SPK from a BDD in our series of 26 pancreas transplants, thus yielding an occurrence rate of 3.8 %, which is relatively low compared with the UNOS data.

A variety of risk factors for graft venous thrombosis in pancreas transplantation have been reported [4–9]. We summarized these reports and show the various risk factors in Table 2. Among the risk factors for venous thrombosis, we emphasize that the three-dimensional positional relationships among the external iliac vessels, pancreas graft, graft artery, portal vein, and anastomosed duodenum and intestine are absolutely critical. In particular, the portal vein must be given special attention to avoid compression by the iliac artery or intestine after positioning of the graft. This is a key point for successful pancreas transplantation, in addition to meticulous vascular anastomoses.

We were unable to perform a statistical analysis because there was only one case of venous thrombosis in our series. There were no differences in the donor- and operation-related factors between the patient with graft venous thrombosis and the others. However, the recipient BMI was higher than the average BMI of the other patients. Obesity may have led to a higher abdominal pressure and caused graft vein compression and congestion, leading to venous thrombosis.

Virchow’s triad has been a major concept considered to lead to thrombus formation [13]. In pancreas transplantation, (1) the blood flow of the splenic and portal veins becomes readily congested owing to the removal of the donor spleen, (2) the vascular endothelium is damaged owing to ischemia/reperfusion injury, and (3) patients with type I diabetes and end-stage renal failure have a tendency to develop a hypercoagulable state [13]. Pancreas transplantation thus fulfills the entire Virchow’s triad. Therefore, some reports recommend continuous intravenous infusion of low-dose heparin or subcutaneous injection of low-molecular-weight heparin to prevent thrombosis [16, 20]. In many pancreas transplant centers, anticoagulation therapy is employed to prevent venous thrombosis starting on the day of the operation. In contrast, we do not generally use anticoagulants to prevent bleeding complications. Although our approach failed in only one patient, the remaining 23 recipients who received whole pancreas grafts had no thrombotic events without heparinization. In addition to graft venous thrombosis, we experienced reoperation for bleeding in the early postoperative stage in three of the 26 patients. The risk of postoperative bleeding is generally high in pancreas transplantation. Because uncontrollable bleeding may become fatal, perioperative anticoagulant therapy should be used only for recipients with a high risk of thrombosis. We start anticoagulant therapy only when signs of venous thrombosis are observed. Thrombosis can be detected in an early stage by frequent and careful examinations using the recently developed, sophisticated ultrasonographic techniques.

Sufficient fluid infusion is a basic policy for the prevention of thrombosis used in our department, because the concentration of hematocrit secondary to dehydration increases the viscosity of the blood and becomes a risk factor for thrombosis [21]. The postoperative hematocrit level should be maintained at approximately 25 %. We administer albumin products aggressively to maintain the intravascular fluid volume. On PODs 1 and 2, an infusion volume exceeding 1000–1500 mL beyond the urine volume was given to the recipients. A sufficient fluid infusion volume, equivalent to the urination volume, was given thereafter, as shown in Fig. 1. The parameters that we check include the body weight, inferior vena cava diameter, and cardiothoracic ratio. We evaluated these parameters carefully and comprehensively to maintain the intravascular volume.

Early detection is extremely important for adequate treatment of graft venous thrombosis. Recipients with complete occlusion of the portal vein reportedly develop hyperglycemia, epigastric pain, and hyperamylasemia [19]. However, based on the experience of Gilbert et al. [19] involving 25 patients with pancreas graft thrombosis, recipients with partial portal vein occlusion often present with no symptoms. Therefore, frequent Doppler ultrasonographic examinations should be performed as a method of early detection of venous thrombosis. According to Gilbert et al. [19], 20 of 25 patients with pancreas graft thrombosis had complete portal vein occlusion. The graft was totally necrotic in 14 of the patients, whereas the other six patients showed partial necrosis of the graft. Although the thrombus was removed with a Fogarty catheter, two of the six patients lost their graft in that study. In three of the other five patients whose graft vein was partially occluded, intravenous administration of urokinase and removal of the thrombus rescued the graft. This confirms that early treatment before complete occlusion of the graft vein is critical.

In conclusion, as the largest series of pancreas transplantation performed so far at a single institution in Japan, our series yielded 10-year patient, pancreas and kidney survival rates of 100, 67 and 68 %, respectively, which are better than the worldwide data reported in the International Pancreas Transplant Registry. Among the 26 pancreas transplantations, there was one graft loss due to graft venous thrombosis in a recipient with a high BMI. Although pancreas transplantation may be complicated by graft thrombosis, routine postoperative anticoagulation therapy is not necessarily required for its prevention if sufficient fluid infusion is strictly controlled and the graft blood flow is frequently monitored. When graft thrombosis occurs, early detection and medical intervention are extremely important before complete occlusion of the portal vein in order to improve the survival of the pancreas graft.

## References

[1] Gruessner AC (2011). 2011 Update on pancreas transplantation: comprehensive trend analysis of 25,000 cases followed up over the course of twenty-four years at the International Pancreas Transplant Registry (IPTR). Rev Diabet Stud.

[2] Ishibashi M, Ito T, Sugitani A, Furukawa H, Sekiguchi S, Gotoh M (2008). Present status of pancreas transplantation in Japan—donation predominantly from marginal donors and modified surgical technique: report of Japan pancreas transplantation registry. Transplant Proc.

[3] Martins L, Henriques AC, Dias L, Pedroso S, Almeida M, Santos J (2011). One hundred eleven simultaneous pancreas–kidney transplantations: 10-year experience from a single center in Portugal. Transplant Proc.

[4] Humar A, Kandaswamy R, Granger D, Gruessner RW, Gruessner AC, Sutherland DE (2000). Decreased surgical risks of pancreas transplantation in the modern era. Ann Surg.

[5] Troppmann C, Gruessner AC, Benedetti E, Papalois BE, Dunn DL, Najarian JS (1996). Vascular graft thrombosis after pancreatic transplantation: univariate and multivariate operative and non-operative risk factor analysis. J Am Coll Surg.

[6] Marques RG, Rogers J, Chavin KD, Baliga PK, Lin A, Emovon O (2004). Does treatment of cadaveric organ donors with desmopressin increase the likelihood of pancreas graft thrombosis? Results of a preliminary study. Transplant Proc.

[7] Muthusamy AS, Vaidya AC, Sinha S, Atabani SF, Haque T, Jones G (2009). Pancreas allograft thrombosis following intravenous immunoglobulin administration to treat parvovirus B19 infection. Transpl Infect Dis.

[8] Humar A, Ramcharan T, Kandaswamy R, Gruessner RW, Gruessner AG, Sutherland DE (2004). The impact of donor obesity on outcomes after cadaver pancreas transplants. Am J Transplant.

[9] Kandaswamy R, Humar A, Gruessner AC, Harmon JV, Granger DK, Lynch S (1999). Vascular graft thrombosis after pancreas transplantation: comparison of the FK 506 and cyclosporine eras. Transplant Proc.

[10] Humar A, Ramcharan T, Kandaswamy R, Gruessner RW, Gruessner AC, Sutherland DE (2004). Technical failures after pancreas transplants: why grafts fail and the risk factors—a multivariate analysis. Transplantation.

[11] Gruessner AC, Sutherland DE (2005). Pancreas transplant outcomes for United States (US) and non-US cases as reported to the United Network for Organ Sharing (UNOS) and the International Pancreas Transplant Registry (IPTR) as of June 2004. Clin Transplant.

[12] Gruessner RW, Dunn DL, Gruessner AC, Matas AJ, Najarian JS, Sutherland DE (1994). Recipient risk factors have an impact on technical failure and patient and graft survival rates in bladder-drained pancreas transplants. Transplantation.

[13] Burke GW, Ciancio G, Figueiro J, Buigas R, Olson L, Roth D (2004). Hypercoagulable state associated with kidney–pancreas transplantation. Thromboelastogram-directed anti-coagulation and implications for future therapy. Clin Transplant.

[14] Benz S, Busing M, Kruger B, Mayer JM, Obermaier R, Keck T (2004). Pancreas graft thrombosis: is there a role for trypsin. Pancreas.

[15] Kapur S, Bonham CA, Dodson SF, Dvorchik I, Corry RJ (1999). Strategies to expand the donor pool for pancreas transplantation. Transplantation.

[16] Schenker P, Vonend O, Ertas N, Wunsch A, Schaeffer M, Rump LC (2009). Incidence of pancreas graft thrombosis using low-molecular-weight heparin. Clin Transplant.

[17] Sollinger HW, Odorico JS, Knechtle SJ, D’Alessandro AM, Kalayoglu M, Pirsch JD (1998). Experience with 500 simultaneous pancreas–kidney transplants. Ann Surg.

[18] Hollinger EF, Powelson JA, Mangus RS, Kazimi MM, Taber TE, Goble ML (2009). Immediate retransplantation for pancreas allograft thrombosis. Am J Transplant.

[19] Gilabert R, Fernandez-Cruz L, Real MI, Ricart MJ, Astudillo E, Montana X (2002). Treatment and outcome of pancreatic venous graft thrombosis after kidney–pancreas transplantation. Br J Surg.

[20] Troppmann C (2010). Complications after pancreas transplantation. Curr Opin Organ Transplant.

[21] Yasaka M, Beppu S (1993). Hypercoagulability in the left atrium: part 2: coagulation factors. J Heart Valve Dis.

